# Neural systems for social cognition: gray matter volume abnormalities in boys at high genetic risk of autism symptoms, and a comparison with idiopathic autism spectrum disorder

**DOI:** 10.1007/s00406-015-0623-z

**Published:** 2015-08-02

**Authors:** Marcia N. Goddard, Hanna Swaab, Serge A. R. B. Rombouts, Sophie van Rijn

**Affiliations:** 1 Department of Clinical Child and Adolescent Studies, Faculty of Social and Behavioural Sciences, Leiden University, Wassenaarseweg 52, 2333 AK Leiden, The Netherlands; 2 Institute of Psychology, Leiden University, Leiden, The Netherlands; 3 Leiden Institute for Brain and Cognition, Leiden, The Netherlands; 4 Department of Radiology, Leiden University Medical Center, Leiden, The Netherlands

**Keywords:** Social cognition, Social behavior, Chromosome anomalies, Autism spectrum disorders, Imaging

## Abstract

Klinefelter syndrome (47, XXY) is associated with several physical, cognitive, and behavioral consequences. In terms of social development, there is an increased risk of autism symptomatology. However, it remains unclear how social deficits are related to abnormal brain development and to what degree underlying mechanisms of social dysfunction in 47, XXY are similar to, or different from, those in idiopathic autism (ASD). This study was aimed at investigating the neural architecture of brain structures related to social information processing in boys with 47, XXY, also in comparison with boys with idiopathic ASD. MRI scans of 16 boys with 47, XXY, 16 with ASD, and 16 nonclinical, male controls were analyzed using voxel-based morphometry (VBM). A region of interest mask containing the superior temporal cortex, amygdala, orbitofrontal cortex (OFC), insular cortex, and medial frontal cortex was used. The Social Responsiveness Scale (SRS) was used to assess degree of autism spectrum symptoms. The 47, XXY group could not be distinguished from the ASD group on mean SRS scores, and their scores were significantly higher than in controls. VBM showed that boys with 47, XXY have significant gray matter volume reductions in the left and right insula, and the left OFC, compared with controls and boys with ASD. Additionally, boys with 47, XXY had significantly less gray matter in the right superior temporal gyrus than controls. These results imply social challenges associated with 47, XXY may be rooted in neural anatomy, and autism symptoms in boys with 47, XXY and boys with ASD might have, at least partially, different underlying etiologies.

## Introduction

Klinefelter syndrome (47, XXY) is a genetic condition in which boys have an additional X chromosome, leading to the 47, XXY chromosomal pattern. Physical consequences include gonadal hormone deficiency, infertility, and long extremities [11]. Many studies have focused on the physical, cognitive, and behavioral consequences of 47, XXY [10, 25, 32]. In recent years, attention has also shifted to the neural basis underlying cognitive and behavioral characteristics of 47, XXY. Structural imaging studies thus far indicate that compared with nonclinical controls, males with 47, XXY have decreased total brain volume, enlarged ventricles, as well as smaller caudate, cerebellar, temporal, and frontal volumes [26, 27, 30, 37, 46, 57]. Other studies have reported regions of decreased gray matter in the amygdala, insular cortex, hippocampus, cingulate, occipital lobe, parietal lobe, temporal pole, inferior frontal lobe, and superior temporal gyrus (STG) in males with 47, XXY [15, 22, 42, 43, 46]. Additionally, functional MRI studies have reported deviant neural activation in the superior temporal gyrus, superior temporal sulcus, supramarginal gyrus, amygdala, insula, fusiform gyrus, and middle frontal gyrus [12, 51, 53].

One would expect that these brain abnormalities give rise to cognitive difficulties such as language and reading disorders, executive dysfunction, and social-cognitive deficits. These problems have indeed been reported in 47, XXY [10, 31, 49, 50, 52, 56]. A few of the main social-cognitive deficits associated with 47, XXY are difficulties in facial affect recognition, identification, and verbalization of emotions, theory of mind, and interpreting tone of voice [49, 50, 56] Such neural and cognitive deficits may help explain an increased risk of developmental psychopathology that is reported in individuals with 47, XXY, including psychotic disorders, bipolar disorder, ADHD, and autism spectrum disorder (ASD) [9, 13, 17, 21, 47, 48]. A formal ASD diagnosis requires clinically significant and persistent deficits in social communication and social interaction, as well as restricted, repetitive patterns of behavior, interests, or activities [6]. However, even in the absence of a formal ASD diagnosis, prominent social and communicative difficulties may be present. Difficulty in social interactions, shyness, social withdrawal, problems with assertiveness, and increased levels of autistic traits (as measured with the Social Responsiveness Scale) [19] have been reported [9, 47, 49, 53, 55]. As the development of social competence during childhood is essential for successful participation in a relatively complex and fast-paced society as an adult, it is important to gain insight into the mechanisms underlying social dysfunction in 47, XXY.

Although there is overlap in social behavioral problems between individuals with 47, XXY and individuals with ASD, recent studies have highlighted variability in the underlying mechanisms driving these problems. For example, boys with 47, XXY show more social anxiety than boys with ASD [55], and they display a specific cluster of autism symptoms that differs from idiopathic ASD in the ability to employ social verbalization/chat, offering to share objects, and interest in other children [14]. Additionally, theory of mind impairments in 47, XXY appear to be related to executive dysfunction, while these impairments appear to be related to language and face recognition problems in boys with ASD [56]. In line with these results, both boys with 47, XXY and boys with ASD show deviations in neural networks associated with facial affect labeling. However, while these deviations are located in the amygdala in boys with ASD, boys with 47, XXY show deviant frontal activation [12]. It is important to also study the underlying mechanisms in terms of brain development. In this respect, it would be relevant to know to what degree neural architecture (in terms of gray matter volume) of structures related to social functioning is affected in boys with 47, XXY, and to assess to what degree morphological deviations are similar to, or different from, those of boys with ASD.

Many different brain structures are involved in decoding social stimuli. For instance, the basic processing of facial information (e.g., gaze shifts and mouth movements, important aspects of emotional expressions) and the recognition of biological motion involve the superior temporal cortex (i.e., both the superior temporal gyrus and sulcus). The amygdala is important for more complex social judgments based on facial information, assessing the significance of social information and recognizing threat or danger [2]. The orbitofrontal cortex (OFC) is involved in many aspects of social competence and social-cognitive information processing including the evaluation of sensory stimuli, reward and punishment-related behavior, social decision making, theory of mind, self-reflection, and the representation of facial expressions and identity [36, 39, 40]. The insular cortex mediates recognition of, and responses to, emotional stimuli, regulation of autonomic states related to emotional processes, as well as the representation of one’s internal state [2]. Lastly, a structure involved in more complex interpretation of social-cognitive stimuli including face familiarity, theory of mind, and the distinction between self and other is the medial frontal cortex [3].

The current study is based on the hypothesis that the anatomical maturation of this network may be adversely affected in boys with an extra X chromosome. Imaging techniques may help uncover different etiologies of risk of social dysfunction. Nevertheless, structural imaging studies focusing on males with 47, XXY are relatively sparse. In contrast, many studies have addressed structural brain abnormalities in regions related to social functioning in individuals with ASD. A recent review evaluated structural imaging studies in ASD. Relevant to the current study, lower gray matter volume in the superior temporal cortex and higher gray matter volume in the medial frontal cortex were systematically reported in boys with ASD compared with nonclinical, age-matched, male controls [18]. However, to date, no studies have assessed regional morphologic brain differences between boys with 47, XXY and boys with ASD, which would be very relevant considering the differences in brain activation during social information processing between boys with 47, XXY and boys with ASD that were reported in a recent fMRI study [12].

The main aim of this study was to assess the neural architecture of brain regions associated with social functioning in boys with 47, XXY and boys with ASD, using voxel-based morphometry. The focus was on the superior temporal cortex, amygdala, OFC, insular cortex, and medial frontal cortex. Significant differences would suggest social dysfunction in Klinefelter syndrome may be anchored in anatomical brain deviations. The secondary aim was to investigate gray matter volumetric differences between boys with 47, XXY and boys with ASD in these structures, to assess whether neural architecture of brain areas important for social information processing is differentially affected.

## Methods

### Participants

Sixteen boys with 47, XXY, 16 boys with ASD, and 16 nonclinical, male controls between the ages of 9 and 18 were included in analyses. Analysis of variance (ANOVA) did not reveal a significant effect of group on age [*F*(2,45) = .573; *p* = .568]. Participants completed the block design and vocabulary subtests of the Dutch adaptations of the Wechsler Scales (WAIS-III and WISC-IV) [58, 59]. The subtest vocabulary measures the degree to which one has learned, is able to comprehend, and verbally expresses vocabulary. The subtest block design measures spatial perception, visual abstract processing, and problem solving. These two subtests form the V-BD short form. The V-BD short form is often used to estimate full scale IQ (FSIQ) according to the algorithm (2.9 × (sum of normed scores) + 42) [16]. The V-BD short form correlates highly with WISC full scale IQ (*r* = .88) [29] and has been found valid for the estimation of intelligence, with good reliability (*r* = .91) and validity (.82) [16]. There was a significant effect of group on IQ [*F*(2,44) = 11.73; *p* < .001], with the 47, XXY (*n* = 15) group having a significantly lower IQ than both controls (*n* = 16) and the ASD group (*n* = 16) (*p* < .001 in both instances). There was also a significant effect of group on internalizing problems [*F*(2,36) = 10.66; *p* < .001], externalizing problems [*F*(2,36) = 9.47, *p* < .001], and total problems [*F*(2,36) = 17.76; *p* < .001] (as measured by the Child Behavior Checklist [1]). The 47, XXY (*n* = 14) and ASD (*n* = 11) groups had significantly more internalizing problems than controls (*n* = 14) (*p* = .001 in both instances), significantly more externalizing problems than controls (*p* = .027 and *p* < .001, respectively), and significantly more total problems than controls (*p* < .001 in both instances). Background variables are summarized in Table 1. In the 47, XXY group, four participants received testosterone replacement therapy at the time of the study, while 12 did not. In the ASD group, none of the participants used psychiatric medication at the time of the study.

**Table 1:** Summary of background variables

Group	µ Age	µ IQ	µ CBCL int.	µ CBCL ext.	µ CBCL tot.
47, XXY	13.20 (2.29)	80 (12.6)	18.64 (8.40)	10.64 (8.59)	55.64 (20.15)
ASD	12.84 (1.74)	102 (16.0)	19.09 (13.84)	15.27 (8.01)	68.09 (34.92)
Controls	12.38 (2.41)	103 (16.1)	4.71 (3.85)	3.43 (2.90)	16.71 (10.48)

*CBCL int.*, total score on “internalizing problems” scale of Child Behavior Checklist; *CBCL ext.,* total score on “externalizing problems” scale of Child Behavior Checklist; *CBCL tot.,* total score on “total problems” scale of Child Behavior Checklist

The 47, XXY group was recruited using various strategies, to avoid recruitment bias as much as possible. The sample consisted of nine children who were actively followed up after prenatal diagnosis with the help of clinical genetics departments in the Netherlands and Belgium, as well as seven children whose parents actively sought information about the condition of their child (recruited through support groups and calls for participants, with the help of the Dutch Klinefelter Association), and those who were seeking help for developmental problems (recruited through pediatricians, psychologists, psychiatrists, and clinical genetics departments). The ASD group was recruited through the Center for Autism, a Pediatric Psychiatric Outpatient Department in the Netherlands. All boys with ASD were classified according to the DSM-IV criteria [5] using the Autism Diagnostic Interview-Revised (ADI-R) [34], parental questionnaires, parental interviews, developmental history and family history, information from primary physicians, as well as elaborate expert clinical observations. All ASD diagnoses were reached through consensus among a multidisciplinary team of mental health professionals, including board-certified pediatric psychiatrists with experience in the field of autism. Nonclinical controls were recruited through schools in the western part of the Netherlands and screened for psychopathology. None scored in the clinical range (>70) on any of the scales of the Child Behavior Checklist (CBCL) [1].

Inclusion criteria for all participants were Dutch as primary language and an age between 10 and 18 years. Exclusion criteria were a recent history of substance abuse, intellectual disability (<60 IQ points), scan or motion artifacts (i.e., mean displacement >5 mm), as well as neurological conditions (e.g., structural brain damage due to prenatal/birth complications, tumors, strokes, or diseases affecting the central nervous system). All participants and their parents received a complete description of the study and provided written informed consent prior to participation, in accordance with the Declaration of Helsinki. All children received a gift card for participation, and travel costs were reimbursed. The experiment was approved by the Ethical Committee of the Leiden University Medical Center, Leiden, the Netherlands.

### Procedure

All scans were administered in one morning or afternoon at the Leiden University Medical Center (Leiden, the Netherlands). Upon arrival, participants were screened for metals or other dangerous physical conditions using the MRI safety check list. Subsequently, they were escorted to the mock scanner, which was used to acclimate participants to the scanner environment. Participants were allowed to spend as much time as needed in the mock scanner. Participants underwent anatomical scanning while watching an animated cartoon.

### MRI data acquisition

Scanning was performed on a 3-T Philips Achieva whole body MRI scanner (Philips Healthcare, Best, the Netherlands), using an eight-channel SENSE receiver head coil. T1-weighted anatomical scans [TR = 9.75 ms, TE = 4.60 ms, flip angle = 8°, 140 transverse slices, 1.167 mm × 1.167 mm × 1.200 mm, FOV = 224.000 × 177.333] were obtained, while participants watched a movie. All anatomical scans were reviewed and cleared by a radiologist. No anomalous findings were reported.

### Outcome measures

#### Autism spectrum symptoms

The Social Responsiveness Scale (SRS) [19] is a 65-item parent-report questionnaire that was used to assess the degree of autism spectrum symptoms. It includes items that ascertain social awareness, social cognition, social communication, social motivation, and autistic mannerisms. Higher scores indicate stronger autism traits. A validation study [20] indicated that the SRS was highly correlated with the Autism Diagnostic Interview-Revised (ADI-R) [34]. Coefficients were higher than .64 between SRS scores and all ADI-R scores. Total SRS scores were used as an indication of autism spectrum symptoms.

#### Voxel-based morphometry

Structural data were analyzed using FSL-VBM [23] (http://fsl.fmrib.ox.ac.uk/fsl/fslwiki/FSLVBM), an optimized VBM protocol [28] carried out with FSL tools [44]. First, structural images were brain-extracted and gray-matter-segmented before being registered to the MNI 152 standard space using nonlinear registration [4]. The resulting images were averaged and flipped along the *x*-axis. The mirror images were then averaged to create a left–right symmetric, study-specific gray matter template. Second, all native gray matter images were nonlinearly registered to this study-specific template and “modulated” to correct for local expansion (or contraction) due to the nonlinear component of the spatial transformation. The modulated gray matter images were then smoothed with an isotropic Gaussian kernel with a sigma of 3 mm. Finally, voxel-wise GLM was applied using permutation-based nonparametric testing, correcting for multiple comparisons across space using threshold-free cluster enhancement (TFCE). Because of the specific hypotheses regarding volumetric differences in brain structures involved in social-cognitive information processing, a region of interest mask consisting of the superior temporal cortex, amygdala, OFC, insular cortex, and medial frontal cortex was used. All groups were mutually compared, meaning six contrasts were set up: 47, XXY < HC, 47, XXY > HC, 47, XXY < ASD, 47, XXY > ASD, ASD < HC, and ASD > HC.

## Results

### Autism spectrum symptoms

SRS total scores were available for 14 boys in the control group [*M*_SRS_ = 23.4 (SD = 12.7)], 13 in the 47, XXY group [*M*_SRS_ = 73.8 (SD = 25.0)], and 15 in the ASD group [*M*_SRS_ = 94.2 (SD = 39.5)]. A significant effect of group on SRS scores was found [*F*(2,37) = 23.59; *p* < .001], for which Bonferroni post hoc testing showed that this was due to significantly higher mean scores in both the 47, XXY (*p* < .001) and ASD (*p* < .001) groups compared with controls. No significant difference in mean scores between the 47, XXY and ASD groups was found.

### Voxel-based morphometry

**Fig. 1:**
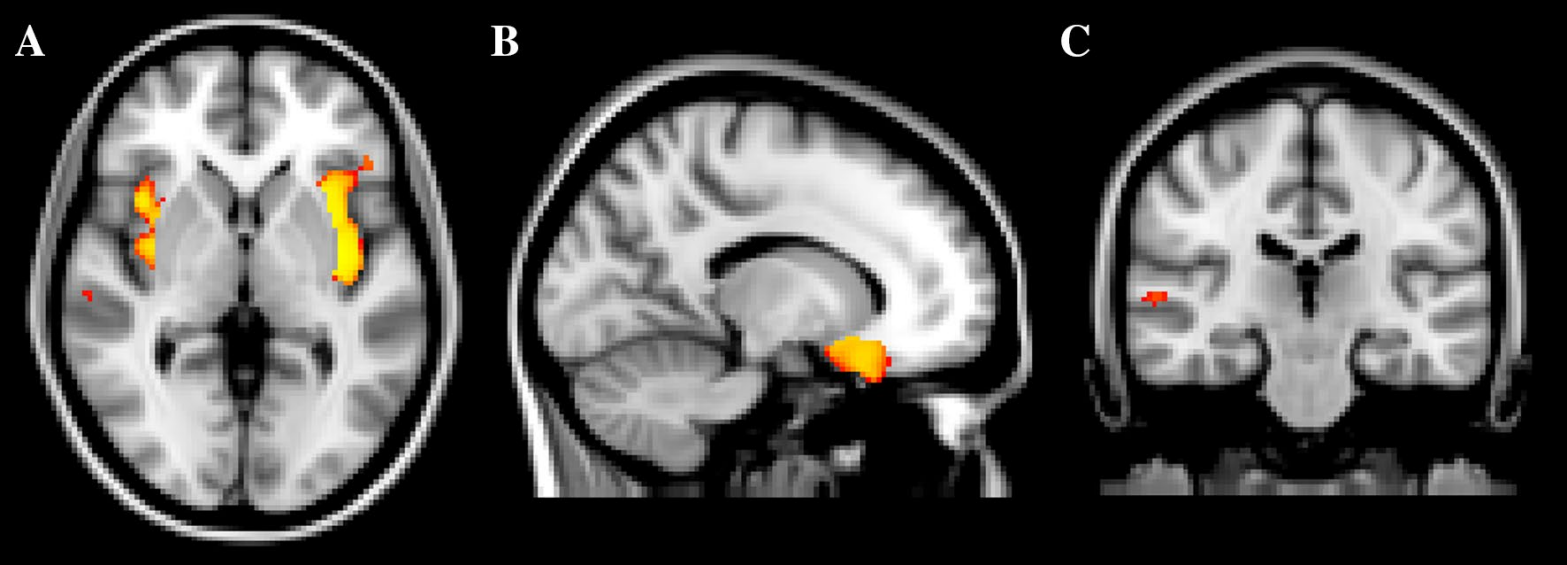
Clusters of significantly deviating gray matter volume. **a** 47, XXY < controls *left* and *right* insular cortices; **b** 47, XXY < controls *left* orbitofrontal cortex, and **c** 47, XXY < controls *right* superior temporal gyrus

**Fig. 2:**
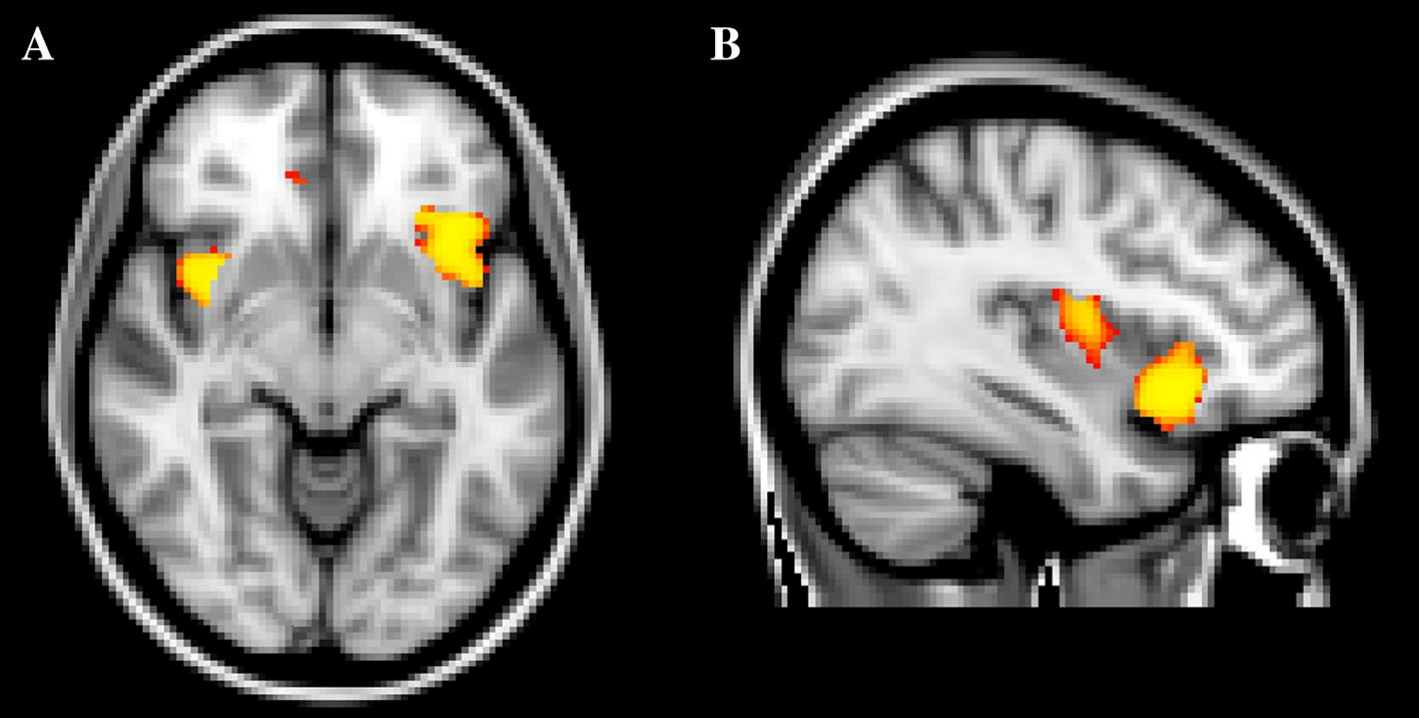
Clusters of significantly deviating gray matter volume. **a** 47, XXY < ASD *left* and *right* insular cortices and **b** 47, XXY < ASD *left* orbitofrontal cortex. *ASD* autism spectrum disorders

Significant TFCE-based thresholding results are summarized in Table 1. The 47, XXY group had significantly less gray matter in the left and right insular cortices (*p* < .001), the left OFC (*p* < .01), and the right STG (*p* < .05), compared with controls (Fig. 1). Additionally, the 47, XXY group had significantly less gray matter in the left (*p* = .001) and right insular cortices (*p* = .002), as well as in the left OFC (*p* = .001), compared with the ASD group (Fig. 2). Other contrasts did not result in significant differences between groups (Table 2).

**Table 2:** Characteristics of clusters of significantly differing gray matter volume

Contrast	No. of voxels	Cluster volume (mm^3^)	Max *t* value	Location
47, XXY < NCC	1414	11,312	5.83	Left insular cortex
	1197	9576	6.02	Right insular cortex
	450	3600	4.01	Left OFC
	26	224	4.39	Right STG
47, XXY < ASD	1429	11,936	5.17	Left OFC
				Left insular cortex
	340	2720	5.64	Right insular cortex

*OFC* orbitofrontal cortex, *STG* superior temporal gyrus, *NCC* nonclinical controls; *ASD* autism spectrum disorder

## Discussion

In this imaging study, the neural architecture of brain regions associated with social functioning, i.e., the superior temporal cortex, amygdala, orbitofrontal cortex (OFC), insular cortex, and medial frontal cortex, was investigated in boys with 47, XXY, compared with boys with ASD and nonclinical, male controls. The results showed that boys with 47, XXY have significant gray matter volume reductions in the left and right insular cortices, as well as the left OFC, compared with healthy controls. These structures were also found to be significantly smaller in boys with 47, XXY than in boys with ASD. Additionally, boys with 47, XXY had significantly smaller gray matter volumes in the right superior temporal gyrus (STG) compared with controls.

In general, the OFC is associated with the evaluation of sensory stimuli, reward and punishment-related behavior, social decision making, and the representation of facial expressions and identity [36, 39, 40]. Importantly, the OFC is also involved in processing mental state concepts and self-reflection [7], related to both theory of mind and emotional literacy. Both theory of mind impairments and alexithymia have been reported in boys with 47, XXY [49, 56]. Although speculative, the OFC volume reductions reported in the current study might be one of the anatomical underpinnings of these problems. Previous studies have not reported volume reductions in the OFC specifically, but reduced gray matter in frontal areas has been found repeatedly [22, 26, 27, 43, 46]. Additionally, one study reported reductions in white matter regions near the OFC in their sample [15].

The insular cortex is involved in recognition of and responses to emotional stimuli, regulation of autonomic states related to emotional processes, as well as the representation of one’s internal state [2]. The finding of deviating gray matter volume in the insular cortex fits with the finding that individuals with 47, XXY show problems in identification and verbalization of emotional states, are more easily emotionally aroused than controls, and are significantly more influenced by their emotional states than controls when making decisions [49, 54]. Another study suggests the insular cortex is functionally involved in decision-making processes [41], and an fMRI study using a social judgment task reported less neural activation in the insula in individuals with 47, XXY [53]. The bilateral reductions in insular cortex volume found in the current study are in line with results from earlier structural studies [15, 42, 43] and might be an anatomical underpinning of these functional findings.

The STG is involved in the processing of facial information, e.g., small changes in facial expression and gaze direction [2]. The finding of reduced right STG volume is in line with earlier studies reporting impairments in face processing in individuals with 47, XXY [49, 53]. Additionally, Wernicke’s area and Heschl’s gyri are part of the Wernicke–Geschwind model of language processing and are located in the STG, making it a key structure in this model [8, 24]. Although language functions are usually located in the left hemisphere, a functional MRI study reported decreased functional asymmetry in the STG during a language task in individuals with 47, XXY [51], due to increased activation in the right hemisphere. This suggests individuals with 47, XXY may have decreased left-sided dominance for language, which could imply the Wernicke–Geschwind model of language processing also involves right-sided brain regions in this condition. The reported deviations in STG function [51] support the suggestion of a relationship between anatomical deviations and altered functionality. Earlier structural MRI studies consistently reported abnormalities in the temporal lobe as a whole in individuals with 47, XXY [26, 27, 30, 46]. In line with other studies [15, 22, 37, 42], the current study suggests these abnormalities may be located specifically in the STG.

There has been much debate within the field of neuroscience about the relationship between brain anatomy and cognitive and behavioral function in general. This debate also extends to the field of 47, XXY, with the largest reported study by Skakkebaek et al. [43] finding no association between specific differences in gray matter volume and the neuropsychological profile of 47, XXY. It is important to continue research in this area, to gain more insight into the impact of individual brain structures on behavioral function. In addition to assessing linear relationships between volume differences in individual brain structures and specific cognitive or behavioral problems, it may also prove essential to focus on neural networks. The deviations found in the current study (that focused on specific regions of interest) may very well be part of a larger network of structural and functional brain deviations that together lead to the cognitive and behavioral issues associated with 47, XXY. Imaging studies like this may have important theoretical implications, as they offer the opportunity for understanding the underlying mechanisms along a gene–brain–behavior pathway. The results are in line with the notion that a high number of X chromosome genes are expressed in the brain [33]. It has been suggested that X chromosome genes are involved in sexually dimorphic brain development [38], making it more likely that individuals with sex chromosome disorders, such as 47, XXY, show deviations in brain anatomy. Another possibility is that these deviations are the result of gene–gene interactions, or hormonal influences on brain development. Future studies focused on these mechanisms may aid in disentangling these complex issues. However, environmental factors may also have a marked influence on shaping the brain [35], stressing the notion that a deterministic approach toward brain development in 47, XXY is not useful. Instead, early positive experiences and interventions (e.g., theory of mind training [45] or social skills training) may have a positive influence on brain development and may even (partially) ameliorate or prevent severe social dysfunction later in development. Neuroimaging may not only help in identifying targets for treatment, but also offer sensitive tools to design and assess the effectiveness of such interventions in 47, XXY.

The secondary aim of this study was to investigate gray matter volumetric differences between boys with 47, XXY and boys with ASD in brain regions associated with social functioning. The SRS scores suggest boys with 47, XXY have increased levels of autism symptoms, and they could not be distinguished from boys with ASD in terms of overall level of these symptoms, but they showed significant reductions in left and right insular cortex volume, as well as the left OFC, compared with boys with ASD. These findings support the notion that the autism spectrum may be extraordinarily heterogeneous. Various neural etiologies may result in similar behavioral symptoms that are classified as autistic. Because boys with 47, XXY represent a genetically homogenous high-risk group for ASD, these findings might help in identifying biomarkers of social dysfunction related to X chromosomal genes, although much more research is needed in this area. The current findings may stimulate such lines of research.

A limitation of the current study was the relatively small sample size. Additionally, the 47, XXY sample appeared to consist of boys with more severe behavioral issues than samples from earlier studies, e.g., Tartaglia et al. [47], as demonstrated by higher scores on the SRS. It is possible that parents of more severely affected boys with 47, XXY were more motivated to participate in neuroimaging research. However, the behavioral characteristics of the current sample were similar to those of the larger cohort from which they were drawn (for an extensive behavioral description of this cohort, see Van Rijn et al. [55]). In addition, there may be cultural differences in the prevalence of behavioral issues associated with 47, XXY, due to differences in mental health care systems, including protocols for interventions or support over the course of development. Voxel-based morphometry has some inherent limitations, e.g., individual differences in cortical folding patterns influencing the results. The results of the current study are in line with those from earlier imaging studies on 47, XXY, with the exception of a lack of significant differences in amygdala volume. These have been reported in earlier neuroimaging studies [43, 46], but were not present in our sample. It is possible this is due to age-related changes in brain morphology. However, the fact that the significant differences that were found in the current study are similar to those from earlier studies in adult individuals with 47, XXY, seems to suggest deviations in brain anatomy develop early and are relatively stable over time. It is therefore possible that differences in imaging analysis methodology or lack of statistical power have led to discrepancies between volume deviations in the amygdala in earlier studies, and a lack of such deviations in the current sample. Furthermore, the lack of significant volumetric differences between the ASD group and controls may imply they were relatively high functioning. IQ scores seem to corroborate this suggestion. A problem inherent to participating in imaging research is that typically, only a selective subgroup of ASD participants (e.g., those with high functioning autism) is included in a study. Additionally, findings regarding structural brain abnormalities in ASD are often conflicting and depend on many (study-specific) factors, such as inclusion and exclusion criteria, MR acquisition parameters, and details of the image processing pipeline [18]. In the current study, small sample size may have played a role, although the 47, XXY group was equally small, but did show significant volume reductions. Lastly, because this study was explorative in nature and the number of contrasts was limited to six, no correction for multiple comparisons was applied. However, all but one significant result would have survived such a correction.

## Conclusion

In sum, the current study suggests boys with 47, XXY with overt social behavioral problems have abnormalities in neural architecture in specific brain regions associated with social functioning, i.e., the insular cortices, OFC, and STG. This implies the X chromosome may significantly impact brain morphology, and the social problems individuals with 47, XXY encounter may be rooted in neural anatomy. Additionally, the differences between boys with 47, XXY and boys with ASD highlight the idea that for understanding developmental risk of social dysfunction in children with 47, XXY, it is important to not only rely on behavioral observations. Brain–behavior relationships are very complex, and social problems may arise as a consequence of different dysfunctions, not only in children with 47, XXY, but possibly also in children with idiopathic ASD. This suggests it is essential to also gain understanding of neuropsychological and neurobiological mechanisms underlying social problems.
